# Hepatocyte organoids and cell transplantation: What the future holds

**DOI:** 10.1038/s12276-021-00579-x

**Published:** 2021-10-18

**Authors:** Weng Chuan Peng, Lianne J. Kraaier, Thomas A. Kluiver

**Affiliations:** Princess Máxima Center for Pediatric Oncology, Heidelberglaan 25, 3584 CS Utrecht, The Netherlands

**Keywords:** Adult stem cells, Self-renewal

## Abstract

Historically, primary hepatocytes have been difficult to expand or maintain in vitro. In this review, we will focus on recent advances in establishing hepatocyte organoids and their potential applications in regenerative medicine. First, we provide a background on the renewal of hepatocytes in the homeostatic as well as the injured liver. Next, we describe strategies for establishing primary hepatocyte organoids derived from either adult or fetal liver based on insights from signaling pathways regulating hepatocyte renewal in vivo. The characteristics of these organoids will be described herein. Notably, hepatocyte organoids can adopt either a proliferative or a metabolic state, depending on the culture conditions. Furthermore, the metabolic gene expression profile can be modulated based on the principles that govern liver zonation. Finally, we discuss the suitability of cell replacement therapy to treat different types of liver diseases and the current state of cell transplantation of in vitro-expanded hepatocytes in mouse models. In addition, we provide insights into how the regenerative microenvironment in the injured host liver may facilitate donor hepatocyte repopulation. In summary, transplantation of in vitro-expanded hepatocytes holds great potential for large-scale clinical application to treat liver diseases.

## Liver transplants: Perfecting laboratory-grown organoids to replace human donors

Improved methods for growing organs from liver cells in the laboratory hold great potential for treating liver diseases through transplantation. Weng Chuan Peng and co-workers at the Princess Máxima Center for Pediatric Oncology in Utrecht, The Netherlands, have reviewed recent advances in growing and maintaining liver cells, or hepatocytes, in vitro so that they form organoids (miniature organs) suitable for cell replacement therapies. Researchers have used three-dimensional cultures to expand adult hepatocytes from healthy mouse livers and added the crucial inflammatory signaling molecule TNF-α to mimic tissue repair processes. The resulting organoids showed extensive engraftment and repopulation in a mouse liver injury model. Challenges remain in culturing human hepatocytes, but recent breakthroughs in culture and gene editing technologies could soon enable transplants of laboratory-grown organoids, resolving the issue of donor shortages.

## Introduction

The expansion and maintenance of primary hepatocytes in vitro while retaining their functional characteristics have been long-standing challenges in the field^1^. Historically, hepatocytes have been cultured in monolayers on collagen gel, but cells quickly lose morphology and the expression of mature hepatic metabolic genes such as cytochromes P450 (CYPs) using this method^2–4^. Various approaches have been employed to promote the viability and function of hepatocytes in vitro, including 2D culture overlaid with Matrigel or collagen gel (termed ‘sandwich culture’)^5^, cocultures with liver nonparenchymal cell types (e.g., Kupffer cells, hepatic stellate cells [HSCs], or liver sinusoidal endothelial cells [LSECs])^6–8^ and aggregation into three-dimensional (3D) spheroids^9–12^. While these approaches have resulted in marked improvements in hepatocyte function and viability, the long-term expansion and maintenance of functional primary hepatocytes remains intractable.

Due to the difficulty in culturing primary hepatocytes, alternative cell sources have been explored, such as the directed differentiation of embryonic stem cells (ESCs) or induced pluripotent stem cells (iPSCs) towards hepatocytes^13–16^. In this context, specification towards the hepatocyte lineage was achieved by employing factors to activate (or inhibit) specific signaling pathways at various time points during culture to mimic in vivo developmental stages^17^. This method allows the generation of large numbers of hepatocyte-like cells. However, the resulting cells often lack functional maturity when compared to their in vivo counterparts^18^.

In addition to ESC/iPSC-derived cultures, major advances have been made in establishing organoids from adult/fetal progenitors or mature cell types derived from healthy primary tissues. In 2009, the Clevers group described the long-term culture of ‘mini-intestines’ established from leucine-rich repeat-containing G-protein coupled receptor 5-positive (LGR5^+^) crypt base columnar cells (CBCs)^19^. Embedded in 3D extracellular matrix, LGR5^+^ CBCs can be coaxed to self-renew and generate various intestinal cell types in the presence of growth factors such as R-spondin (RSPO), epidermal growth factor (EGF) and noggin (NOG) while spontaneously organizing into crypt-villus structures. Organoid technology has since enabled the in vitro self-renewal of various epithelial cell types, which can be indefinitely expanded while spontaneously self-organizing into 3D structures that resemble their native counterparts^16^. Huch and colleagues described the culture of liver organoids derived from intrahepatic cholangiocytes, i.e., the epithelial cells that form the bile ducts^20,21^. Since then, other groups have shown that organoids can be derived from common bile ducts and gall bladder^22–24^. However, the 3D culture of primary hepatocytes, which are the principal epithelial cell type that performs most liver metabolic functions, remains elusive. Importantly, the ability to expand primary hepatocytes in vitro is a crucial step towards enabling cell transplantation to treat liver diseases. In a recent breakthrough, the Nusse and Clevers groups described the long-term organoid culture of murine and human primary hepatocytes^25,26^. In this review, we will focus on strategies to derive organoids from primary hepatocytes, drawing insights from signals regulating liver homeostasis and regeneration, on the characteristics of these organoids from the perspective of liver physiology, and finally on the application of hepatocyte organoids for cell transplantation.

## Liver homeostasis and repair: insights into culturing hepatocytes in vitro

**Fig. 1:**
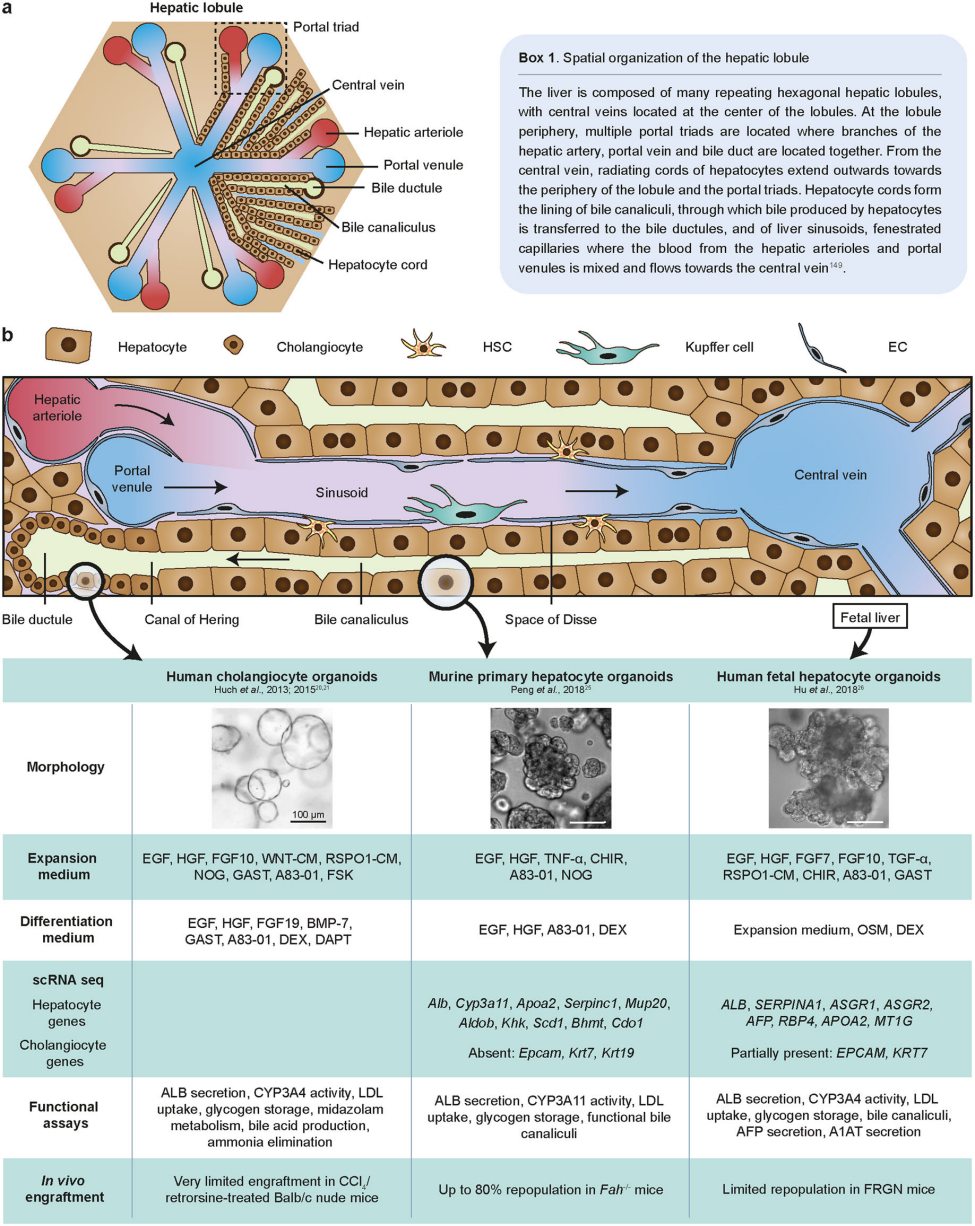
An overview of the liver lobule and characteristics of organoids derived from either hepatocytes or cholangiocytes. **a** Schematic diagram of the hepatic lobule showing the portal triad, central vein, and hepatic cords (see also **Box 1**^149^). **b** A detailed view of the hepatic lobule showing various cell types in the liver (upper panel) and characteristics of cholangiocyte and hepatocyte organoids derived from either adult or fetal liver^20,21,25,26^ (lower panel). Scale bars: 100 µm; CM: conditioned medium; HSC: hepatic stellate cell; EC: endothelial cell.

The liver is essential for processes such as metabolism, drug detoxification and plasma protein production. The two major parenchymal cell types in the liver are hepatocytes and biliary epithelial cells (BECs; cholangiocytes). Hepatocytes perform most of the liver functions, while bile ducts formed by cholangiocytes transport bile acid produced by hepatocytes to the gallbladder. For an overview of liver architecture, see Fig. 1. The liver is a largely quiescent organ, and hepatocytes are long-lived with an estimated lifespan of 200–400 days in rodents^27–29^. In addition to diploid hepatocytes, the liver also contains binucleated and polypoid hepatocytes, which appear during postnatal development, increase with aging and are associated with reduced proliferative capacity^30^. Due to its low physiological turnover, the liver does not utilize a dedicated stem cell compartment, akin to the intestinal crypt, to produce new hepatocytes. Rather, new hepatocytes are generated by the existing pool of mature hepatocytes^31^. However, it remains unclear whether all hepatocytes are capable of self-renewal or if specific subsets of hepatocytes are endowed with unique capabilities to divide. Key questions remain: what are the niche signals regulating hepatocyte homeostatic turnover, and what are the defining characteristics of these hepatocytes?

Recent studies employing lineage tracing in mice offer new perspectives on the turnover of hepatocytes in the resting liver. Wang et al.^32^ observed that hepatocytes adjacent to the central vein (CV), under the influence of WNT signals (WNT9B and RSPO3) secreted by the endothelial cells of the CV, preferentially self-renewed and replaced other hepatocytes in the lobule^32–34^. Hepatocytes around the CV expressed WNT target gene axis inhibition protein 2 (*Axin2*) and progenitor marker T-box transcription factor 3 (*Tbx3*), which are normally expressed during fetal liver development^32^. These pericentral hepatocytes are predominantly diploid but can give rise to daughter cells that become polyploid. Over the course of one year of tracing, 20–30% of the lobule is replaced by daughter cells produced by pericentral hepatocytes. While WNT signaling is predominantly restricted to pericentral hepatocytes in the homeostatic liver, following carbon tetrachloride (CCl_4_)-induced liver injury of the pericentral zone, pathway activation is also observed in midlobular hepatocytes surrounding the injury area^35^. It is perhaps unsurprising that the WNT pathway is implicated in hepatocyte proliferation, given its role in liver development and regeneration^34,36,37^, childhood liver tumors^38,39^ (~80% of hepatoblastoma tumors have activating mutations in *CTNNB1*, encoding β-catenin), and in promoting the stem-cell activity of various tissues^40,41^.

In another study, Lin et al.^42^ identified a subset of hepatocytes that expressed high levels of telomerase and were broadly distributed throughout the lobule. In this study, telomerase reverse transcriptase (TERT)-high cells gave rise to small clones (up to eight cells) after 6 months of lineage tracing in the healthy mouse liver^42^. An interesting aspect of TERT-high cells is that they have reduced metabolic activity, suggesting that they may be somewhat adapted for proliferation rather than for regular hepatocyte function. Interestingly, *TERT* promoter mutations constitute the most frequent genetic alterations in hepatocellular carcinoma, with an overall frequency of 60%^43^. Of note, several recent studies^44–47^ using various lineage tracing models have argued against the notion that hepatocyte renewal is preferentially localized to either the central or portal zone^32,48^ but rather is distributed broadly across the lobule. It is also conceivable that multiple and possibly redundant populations of hepatocytes are capable of self-renewal, since the liver is susceptible to various types of damage differently affecting cells in different parts of the lobule^49^.

A recent study of human tissues using retrospective ^14^C birth dating of cells found that human hepatocytes continuously self-renew during the lifetime of an adult to maintain a relatively young liver^50^. Both diploid and polyploid hepatocytes are capable of self-renewal; however, diploid cells have major advantages over polyploid cells in long-term proliferation^50^. This study on human liver tissues is likely more reflective of physiological settings (e.g., regular exposure to chemicals, pathogens, and drug-induced injury) compared to standard laboratory conditions under which mice are maintained. Nonetheless, the above studies in mice and human tissues provide emerging evidence that hepatocytes undergo homeostatic renewal at a more significant rate than previously thought, that hepatocyte proliferation is associated with low ploidy and low metabolic state and that WNT signaling is a likely pathway mediating homeostatic renewal.

## Liver regeneration

In contrast to the low turnover in the resting state, the liver has a unique regenerative capacity, which is crucial for maintaining liver mass following physical or chemical damage^31^. The events that occur after partial hepatectomy (PHx) in rodents have been well described and serve as a basis for understanding the general principles of liver regeneration^51,52^. Notably, regeneration following PHx is not dependent on a small subset of stem cells. Instead, the majority of hepatocytes are able to proliferate^53^.

### Regenerative signals

Following PHx, the plasma levels of EGF, insulin and glucagon (secreted by the intestine, spleen, and pancreas) rise immediately^54,55^. Hepatocyte growth factor (HGF) pro-peptides are released from the extracellular matrix and cleaved by urokinase-type plasminogen activator (uPA)^56^. Together, these molecules act as potent mitogens to promote DNA synthesis. WNT/β-catenin signaling is another crucial regulator of liver regeneration, which regulates cell proliferation by promoting G1 phase through cyclin D1 and mitosis^57^. Based on our current understanding of WNT pathway regulation, the LGR5/RSPO/zinc and ring finger 3 (ZNRF3) module is likely required for amplifying WNT signals^58,59^. Indeed, this was confirmed by a recent study showing impaired regeneration of the liver in LGR4/5-deficient mice after PHx^34^. LSECs are the likely source of WNT and RSPO^34,60^. Separately, inflammatory cytokines constitute important early signals that initiate liver regeneration^55^. Two major cytokines that are commonly involved in tissue repair processes across many tissue types are tumor necrosis factor alpha (TNF-α) and interleukin 6 (IL-6)^61^. TNF-α is produced by liver and spleen macrophages in response to lipopolysaccharide^62^, while IL-6 is secreted by liver macrophages and hepatocytes as a result of TNF-α activation^63^. Other signals that have been implicated in liver regeneration include fibroblast growth factors 7 (FGF-7)^64^ and 15 (FGF-15; FGF-19 in humans)^65^ and bone morphogenetic protein 7 (BMP-7)^66^. There is likely a redundancy between different signaling pathways, and not all components have been identified^67^.

### Intracellular events

In response to regenerative stimuli, various signaling pathways are activated in hepatocytes that lead to cell cycle progression, DNA synthesis and eventually cell division. Intracellularly, the β-catenin and NOTCH intracellular domains (NICD) translocate to hepatocyte nuclei within minutes after PHx. Cytokine-induced transcription factors, such as signal transducer and activator of transcription 3 (STAT-3), nuclear factor kappa-light-chain-enhancer of activated B cells (NF-κB) and activator protein 1 (AP-1), are activated shortly after^53^. Yes-associated protein 1 (YAP1), a critical organ size regulator, is also nuclearly localized^68^. Although no neighboring cells are lost in PHx, the change in shear stress due to increased blood flow activates YAP1 through the Hippo pathway^69^. Hepatocytes secrete various autocrine and paracrine factors, e.g., vascular endothelial growth factor (VEGF), angiopoietin 1 and 2, platelet-derived growth factor (PDGF), FGF-1 and -2, transforming growth factor alpha (TGF-α) and granulocyte-macrophage colony-stimulating factor (GM-CSF), to promote angiogenesis and stimulate the growth of other liver cell types (HSCs, Kupffer cells and biliary cells)^63^. A change in metabolic profile is also observed as a consequence of regeneration. The expression of genes such as alpha-fetoprotein (AFP), hexokinase and fetal isoforms of aldolase and pyruvate kinase, typically absent in the healthy liver, leads to the acquisition of a fetal-like phenotype in the regenerating liver^70,71^.

### Termination signals

When the normal liver mass has been restored, regeneration is gradually terminated. Transforming growth factor-beta (TGF-β) family members, secreted by Kupffer cells and HSCs, act as a brake for hepatocyte proliferation^67^. Another crucial dampening signal is the suppressor of cytokine signaling (SOCS), a negative regulator of various cytokines. In particular, SOCS-1 and SOCS-3, feedback inhibitors of the interleukin (IL)/Janus kinase (JAK)/STAT-3 pathway, inactivate STAT-3 to terminate hepatocyte proliferation^72,73^. Other cytokines, such as interferon-gamma (IFN-γ), have also been shown to downregulate hepatocyte proliferation^74^. Overall, the study of liver regeneration over the years has provided important insights into signals that both activate and terminate hepatocyte proliferation. How hepatocytes reestablish the metabolic gene expression profile of the homeostatic liver upon termination of regeneration remains unclear.

## Regenerative signals for expanding hepatocytes in vitro

Cholangiocyte organoids have been successfully derived from intra- and extrahepatic cholangiocytes as well as the gallbladder by using combinations of EGF, FGF10, HGF, WNT agonist (WNT3A or CHIR99021), RSPO1, gastrin (GAST), NOG, prostaglandin E2 (PGE2), dexamethasone (DEX), dickkopf-related protein 1 (DKK-1), forskolin (FSK), TGF-β inhibitor (A83-01) and rho-associated protein kinase (ROCK) inhibitor (Y27632)^20–24^. A related cocktail employing EGF, CHIR99021, A83-01, and Y27632 was able to expand murine primary hepatocytes in a 2D monolayer^75^. Hepatocellular carcinoma tumor organoid cultures have also been established utilizing the cholangiocyte organoid medium defined by Huch et al.^21,76^. However, such culture media have thus far failed to establish primary hepatocyte organoids, prompting us to hypothesize that the media may lack crucial signals for this purpose.

Drawing insights from liver regeneration in vivo, we speculated that inflammatory signals may be a crucial factor for initiating hepatocyte proliferation in vitro. Indeed, culture medium containing TNF-α, along with EGF, HGF, CHIR99021, A83-01, and Y27632, enabled robust proliferation of murine primary hepatocytes^25^. Using this protocol, approximately 15% of plated hepatocytes were able to form organoids. In the first week of culture, expanded hepatocytes formed small spheroids, which gradually expanded up to several hundred micrometers in the first 3 weeks to form rosette-like structures (Fig. 1b). Notably, they are morphologically distinct from the ‘cystic’ structures typically observed in cholangiocyte organoids described by Huch et al. and others (Fig. 1b)^20,21,23,24^. While mature hepatocytes are predominantly binucleated or polyploid, we noticed that in vitro-expanded hepatocytes are mostly mononucleated and have a high nucleus-to-cytoplasm ratio, reminiscent of regenerating hepatocytes in the liver following injury^25,77^. The hepatocyte-specific marker hepatocyte nuclear factor 4 alpha (HNF-4α) was broadly expressed, and bile canaliculi structures could be detected, reminiscent of the structural polarity typically observed in the liver lobule. Functionally, hepatocyte organoids retain key hepatic functions, such as albumin (ALB) secretion, low-density lipoprotein (LDL) uptake, glycogen storage, and CYP3A11 enzymatic activity, and contain functional bile canaliculi. Furthermore, hepatocyte organoids could upregulate various metabolic genes under appropriate culture conditions. Remarkably, single-cell RNA sequencing (scRNA-seq) showed that *Alb* and *Cyp3a11* could be induced to levels comparable to murine primary hepatocytes, indicating that the differentiation capacity is retained even after long-term culture. In vivo, hepatocyte organoids could engraft the injured liver of mice with high efficiency and reestablish the expression profile of zonated markers. Finally, hepatocyte organoids can be serially passaged and expanded for at least 8 months (the longest culture was cryopreserved after approximately 1 year in culture, unpublished data). To our knowledge, this is the first study demonstrating long-term 3D culture of hepatocyte organoids that retain key functional characteristics and in vivo regenerative capacity.

## Cytokine dependency and mode of action

Intriguingly, we found that TNF-α is a crucial factor for the establishment of hepatocyte organoid culture^25^. Substituting TNF-α with IL-6, another inflammatory cytokine, did not yield any organoids. Furthermore, the long-term expansion of hepatocytes is continuously dependent on TNF-α. At any point in culture, removal of TNF-α resulted in limited growth of hepatocytes and eventually led to culture deterioration. In vivo, TNF-α is generally considered a crucial signal in liver regeneration. For instance, antibody blockade of TNF-α impaired regeneration^78^, while TNF receptor 1 knockout mice have delayed regeneration^79,80^. The critical role of TNF-α in maintaining in vitro culture is therefore not unexpected.

Earlier hepatocyte culture studies offer some insights into the role of TNF-α in hepatocyte proliferation. A study found that while EGF alone did not promote hepatocyte proliferation in culture, the combination of EGF and TNF-α stimulates proliferation in ~35% of cultured hepatocytes^81^ by mediating cell entry into S phase through cyclin D1 and cyclin-dependent kinase (CDK) 1 and 2. In addition, TNF-α suppressed fetal hepatocyte maturation induced by oncostatin M (OSM) to promote cell cycle progression in culture^82^. TNF-α is also known to prevent apoptosis and promote cell survival and proliferation through NF-κB signaling^83,84^. Indeed, inhibition of NF-κB resulted in culture deterioration^25^, indicating that TNF-α signaling is likely mediated by the NF-κB pathway. Importantly, significant crosstalk exists between the NF-κB and WNT signaling pathways^85^.

A recent study from the Diehl laboratory showed that inflammatory cytokines such as TNF-α can reprogram mature hepatocytes into proliferative hepatocytes with a fetal-like metabolic profile in patients with severe alcoholic hepatitis and in mouse models with liver injury^86^. The acquisition of a fetal profile is mediated by the suppression of the epithelial splicing regulatory protein 2 (ESRP2)-mediated adult splicing program, resulting in the accumulation of fetal splicing variants of genes such as *NF2* and *CSNK1D*, which have reduced kinase activity for YAP1 degradation. Consequently, YAP1 target genes such as *AREG, CTGF*, and *PTGS2* were upregulated. Consistent with in vivo observations, qRT-PCR and splice isoform analysis of hepatocyte organoids cultured in TNF-α medium^25^ revealed significant downregulation of *Esrp2*, accumulation of fetal RNA-splicing variants (*Nf2*, *Slk*, *Fln*, and *Kras*) and upregulation of the aforementioned YAP1 target genes^25^. Importantly, upon TNF-α withdrawal, hepatocyte organoids reestablish *Esrp2* expression and upregulate adult liver-specific markers such as ammonia detoxification pathways and blood coagulation factors. In sum, this study provides a potential mechanism through which TNF-α reprograms adult hepatocytes into fetal-like hepatocytes^86^.

## Cytokine-induced in vitro regeneration beyond the liver

The liver is not the only organ that is dependent on inflammatory signals for regeneration after injury. Indeed, across multiple tissues, for example, skeletal muscle, intestine, colon, hair follicles, skin, and the central nervous system, inflammation and regeneration are tightly coupled^87,88^. The inflammatory cytokines TNF-α and interleukins trigger regenerative responses in adult progenitors or mature cells by activating a multitude of transcription factors (e.g., NF-κB, JAK/STAT, AP-1, YAP1, and NOTCH), thereby activating a transcriptional program that promotes cell survival, proliferation, dedifferentation and acquisition of a fetal-like phenotype^88^. Such observations led us to postulate that regenerative cytokines may have a more general role in cell cycle regulation and could potentially be harnessed beyond the context of injury to expand otherwise quiescent/slow-cycling primary cell types in vitro. Indeed, the regenerative effect of cytokines in vitro has also been employed to expand other tissue types, i.e., muscle and lung, both of which are known to undergo cytokine-driven regeneration following injury^89–91^. Indeed, muscle stem cells (satellite cells) can be expanded long-term with a combination of cytokines (TNF-α, IL-1α, IL-13, and IFN-γ), while lung alveolar type 2 epithelial cells robustly proliferate in the presence of IL-1 and TNF-α. Collectively, these studies provide emerging evidence supporting the role of cytokines in the in vitro culture of otherwise ‘hard-to-culture’ cell types.

## An alternative strategy: fetal-derived human hepatocytes

In a parallel study by the Clevers group, Hu et al.^26^ expanded murine hepatocytes using a cocktail that included EGF, HGF, FGF7 and 10, WNT agonists (CHIR99021 and RSPO1), A83-01 and Y27632 (Fig. 1b). FGF7 is a niche factor previously found to be important for liver regeneration^92^. Interestingly, Hu et al.^26^ observed that cells around the CV have a slightly higher organoid-forming efficiency than the rest of the hepatocytes in the lobule. Diploid and tetraploid hepatocytes, but not octaploid cells, can form organoids. Morphologically, hepatocyte organoids resemble ‘grape-like’ clusters of cells, as opposed to the more densely packed structures observed in hepatocyte organoids established by Peng et al.^25^. Importantly, hepatocyte organoids were able to perform major functions, such as ALB secretion, CYP1A2 activity, glycogen storage and LDL uptake, and displayed extensive bile canaliculi networks. While the cocktail media was able to support organoid establishment, only a small fraction of plated cells formed hepatocyte organoids (0.5–1%). The rate of organoid formation in this medium is considerably lower than that in media with TNF-α. Notably, organoids could be maintained for up to 3 months and showed limited growth afterwards^26^.

To culture human hepatocytes, Hu et al.^26^ adopted a different strategy by using fetal-derived liver cells, which are expected to have higher proliferative capacities than mature hepatocytes and hence are more suitable for long-term propagation. Previous studies indicated that fetal hepatocytes could be maintained in culture for several months while acquiring mature hepatic function^93,94^. In the current study, fetal liver cells were obtained from donor fetuses at 11-20 weeks of gestation using a two-step perfusion method that typically enriches hepatocytes in the adult liver. Approximately 1% of plated cells were able to form organoids^26^. The identity of the cells (or their corresponding developmental stages) that gave rise to organoids was not determined in this study. Using a medium similar to that of murine organoid culture, human fetal liver-derived hepatocyte organoids could be expanded for close to a year. Transmission microscopic imaging showed the accumulation of glycogen particles and revealed cellular features (large nuclei with prominent nucleoli, large numbers of mitochondria with few cisternae, bile canaliculi, tight junctions, etc.) that closely resemble hepatocytes, indicative of maturation in vitro^26^.

## Adult human hepatocyte culture remains challenging

In the same study, Hu et al.^26^ established hepatocyte organoids using human hepatocytes from adult and pediatric sources. However, when compared to fetal-derived hepatocytes or adult mouse primary hepatocytes, the proliferative capacity of mature hepatocytes appeared limited (2-2.5 months). Since the publication of our work, several groups have reported monolayer cultures of human hepatocytes^95–100^. Zhang et al.^96^ found that the expression levels of senescence and cell cycle arrest genes increased markedly during culture, and the growth of adult hepatocytes was arrested after 4 passages (which corresponds to ~300-fold expansion). To counter this cellular senescence effect, cells were cultured under hypoxic conditions and consequently showed a 10,000-fold expansion (up to 8 passages). Separately, Katsuda et al.^100^ attempted to reprogram adult human hepatocytes based on their previously described small-molecule-mediated culture method. They found that adult human hepatocytes showed very limited expansion. Next, Katsuda et al. focused on infant hepatocytes, which have been shown to be easier to expand in culture^101^. Upon treatment with the maturation-inducing factors OSM and DEX, these cells could be differentiated into cells expressing several functional CYP enzymes (e.g., CYP1A2, CYP2B6, and CYP3A4) at levels comparable to mature hepatocytes. Furthermore, they could be passaged for over 10 passages, although the expression of hepatic markers and functional activities (such as CYP enzymatic activities) gradually declined during culture, as noted by the authors. These studies, as well as other recent studies, highlighted the challenges in expanding adult human hepatocytes long-term^95–100^.

## Cell identity and state: bipotent or not?

The identity of cultured hepatocytes in vitro has always been an intriguing question, since earlier studies found that hepatocytes can adopt a biliary fate following injury and in vitro culture^67,102^. The first evidence for hepatocyte to cholangiocyte transition in vivo was shown by Michalopoulos et al.^103^, who demonstrated that dipeptidyl peptidase IV (DPPIV)^+^-transplanted hepatocytes acquired a ductular morphology upon induction of bile duct injury in the DPPIV^–^ host rat model. In a later study, Yanger et al.^104^ used lineage tracing in mouse cholestatic liver injury models to demonstrate that hepatocytes can undergo transition into ductal-like cells upon cholangiocyte damage. Moreover, Schaub et al.^105^ demonstrated that in a mouse model of human Alagille syndrome, a disease characterized by impaired formation of peripheral bile ducts, a partially functional biliary tree is still formed. The researchers used lineage tracing to show that these ducts were of hepatocyte origin and that hepatocytes were capable of *de novo* bile duct formation in this disease model. Together, these studies demonstrate the capability of hepatocytes to transition to a ductal state when cholangiocyte proliferation is suppressed.

Single-cell RNA-seq allows for the evaluation of the heterogeneity among cultured hepatocytes. Based on scRNA-seq, Hu et al.^26^ identified five distinct clusters of cells in their murine hepatocyte cultures, which they defined as noncycling mature hepatocytes (*Alb*^high^), cycling progenitor cells with high levels of progenitor markers (secreted phosphoprotein 1 [*Spp1*]^high^, *Alb*^low^), more primitive progenitor cells with high levels of cycling genes and lacking mature markers (two clusters), and some cells with biliary markers (keratin 7 [*Krt7*]^+^). Consistent with the transcriptomic data, cystic organoids that resemble biliary organoids formed spontaneously in culture from ALB^+^-labeled cells. Furthermore, hepatocytes cultured in biliary media strongly upregulate archetypal biliary markers (KRT7 and KRT19), while hepatocyte markers (HNF-4α and ALB) are repressed.

The hepatocyte to biliary conversion is consistent with previous studies in monolayer culture^75,102^. Using small molecules (CHIR99021, Y27632 and A83-01), Katsuda et al.^75^ demonstrated that mature hepatocytes can be reprogrammed into bipotent progenitors and can be induced to upregulate either hepatocyte or biliary markers. Under culture conditions promoting biliary fate, biliary markers such as KRT19, aquaporins (AQPs) 1 and 9, and ion channel proteins cystic fibrosis transmembrane conductance regulator (CFTR) and anion exchange protein 2 (AE2) were strongly induced. The resulting ductal-like structures secrete fluorescein dye into the luminal space and can respond to the hormone secretin by enlarging their luminal space, indicative of functional BECs.

Separately, scRNA-seq by Peng et al.^25^ revealed broad expression of hepatocyte-specific markers (e.g., *Hnf-4α*, *Alb*, apolipoprotein A1 (*Apoa1*), transthyretin (*Ttr*), and *Serpina1c*) across the entire population of cultured hepatocytes. Cultured cells were enriched in genes that are related to cytolysis, lipoprotein particle remodeling, blood coagulation, retinol metabolic process, regulation of G1/S phase transition, and response to tumor necrosis factor. In addition, a subset of cells highly expressed genes that are related to mitosis, cell cycle regulation, chromosome segregation, remodeling and DNA replication, indicative of cycling hepatocytes. One major difference from other in vitro cultures is the absence of canonical biliary markers (such as epithelial cell adhesion molecule [*Epcam*], *Krt7*, *Krt19*, *Aqp1*, *Aqp4* and claudin 7 [*Cldn7*]), indicating that the vast majority of cells cultured with TNF-α are hepatocytes, without evidence of transdifferentiation. Taken together, the above studies demonstrated that hepatocytes in culture display cellular plasticity, and the biliary fate can be suppressed by adding appropriate signals, yielding solely hepatocytes.

## ‘Proliferative’ vs ‘metabolic’ states

An interesting aspect of hepatocyte organoids is that in vitro culture recapitulates some aspects of liver regeneration^25,26^. This is supported by RNA-seq data comparing hepatocyte organoids with hepatocytes isolated from livers subjected to PHx^26^. We noted that transcription factors typically associated with tissue regeneration and previously described to be critical for liver regeneration after PHx were upregulated ex vivo^88,106,107^. For instance, the transcription factors *Rela* (NF-κB p65 subunit), *Stat3*, *Yap1, Myc, Jun* and *Fos* (which form the AP-1 complex) were detected in organoids but only at low levels or absent in primary hepatocytes isolated from healthy mouse livers. Acute phase proteins, as well as cytokines and chemokines associated with inflammatory responses, were highly expressed in proliferating hepatocytes in vitro. In addition, hepatocyte organoids secrete growth factors, including insulin-like growth factor 2 (IGF2), TGF-α and angiogenic factor VEGF-A^108–110^.

**Fig. 2:**
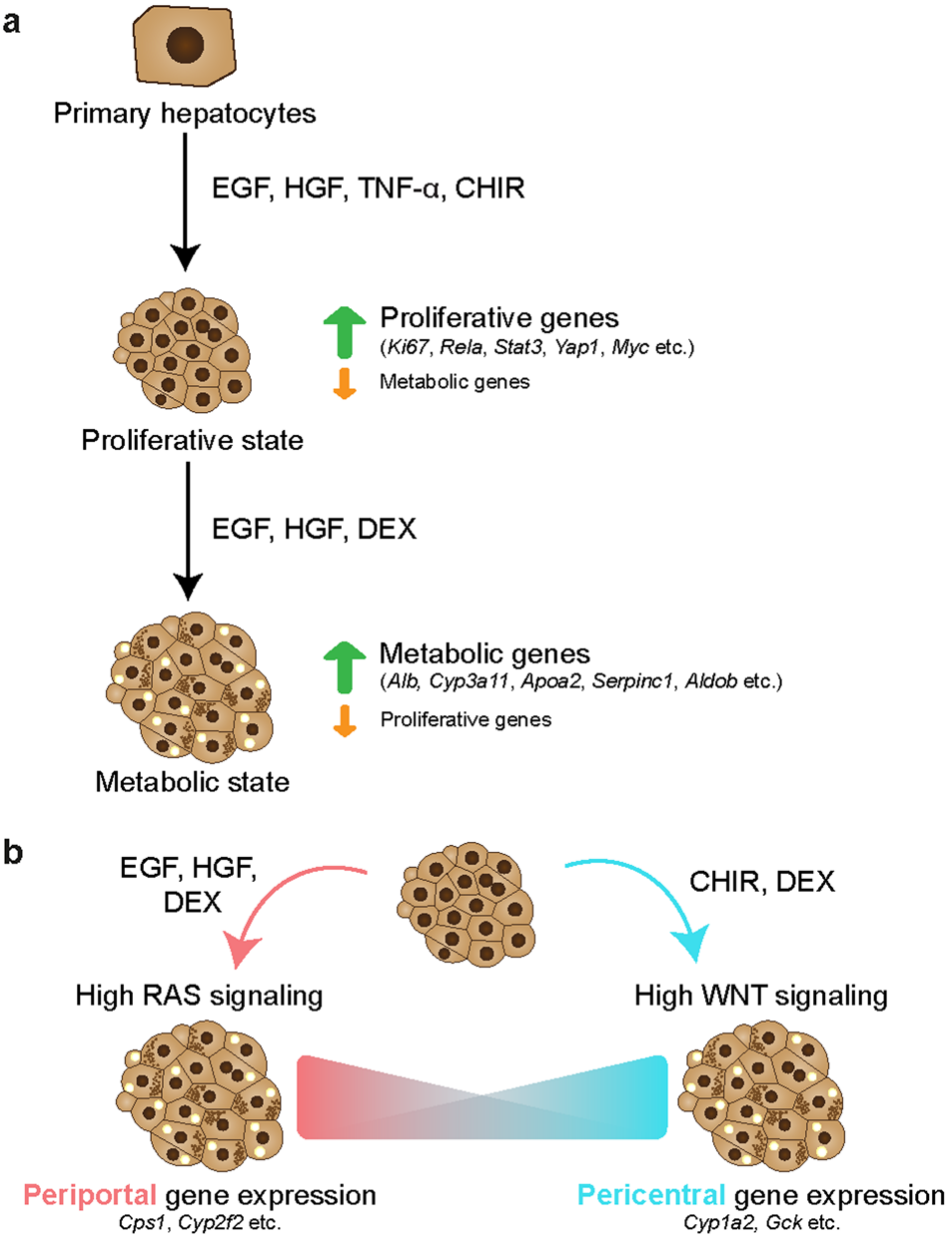
The different states of hepatocyte organoids and modulation of metabolic gene expression in vitro based on the principles of liver zonation. **a** Hepatocyte organoids can adopt a proliferative state in the presence of growth factors or a metabolic state in the presence of maturation factors. **b** Liver zonation is governed by multiple factors, including morphogen gradients. For instance, RAS signaling is high in periportal hepatocytes, while WNT signaling is high in pericentral hepatocytes in vivo. The metabolic gene expression of hepatocyte organoids can be modulated to some extent by either inducing EGF signaling for periportal-like or WNT signaling for pericentral-like gene expression in vitro.

A major consequence of ‘in vitro regeneration’ is the downregulation of metabolic genes that are typically abundantly expressed in the liver, suggesting an adaptation to a ‘proliferative’ state (Fig. 2a). For instance, CYP enzymes (*Cyp3a11* and *Cyp2e1*), apolipoproteins (*Apoa2* and *Apoc3*), serine protease inhibitors (*Serpin1e* and *Serpin3k*) and major urinary proteins (*Mup3* and *Mup20*) were expressed at low levels compared to adult murine hepatocytes^25^. To test whether proliferating hepatocytes reexpress functional genes upon withdrawal of expansion and inflammatory factors, WNT and TNF-α were withdrawn from the culture medium, and DEX was added to promote hepatocyte maturation (see also the section below). Indeed, when hepatocytes cultured in expansion medium (for 2–3 months) were switched to differentiation medium for 3–5 days, cultured cells upregulated suites of genes that correspond to mature hepatocyte functions. scRNA-seq showed that hepatocyte markers such as *Alb* (the most abundant plasma protein), *Cyp3a11*, *Serpina1*, *Aldob, Khk*, *Bhmt* and *Mup20* were induced to levels comparable to those in primary hepatocytes. In addition, functional assays, such as albumin secretion and CYP3A11 enzymatic activity, performed on hepatocyte organoids (at 3–7 months in culture) corroborated the scRNA-seq data. Concomitantly, markers that are associated with regeneration and proliferation were downregulated, correlating with reduced proliferation of organoids in the differentiation medium^25^.

A recent single-cell profiling study surveying the transcriptomic landscape of hepatocytes following PHx provides evidence that hepatocytes transiently reactivate a postnatal-like transcriptomic program to undergo an intense phase of proliferation, with a distinct subset of cells becoming ‘metabolically hyperactive’ to compensate for any temporary deficits in liver function^111^. This division of labor ensures that the liver continues to function optimally while undergoing tissue repair. Taken together, these studies show that hepatocytes can adopt either a ‘proliferative state’ or ‘metabolic state’, both in vivo and in vitro (Fig. 2a). More importantly, hepatocyte organoid cultures retain the ability to upregulate the expression of various metabolic markers typically seen in mature hepatocytes. However, it should be noted that the metabolic profile of the adult liver is highly zonated and regulated by multiple factors, many of which are not present in the current culture conditions^25,26,75^. In the following section, we discuss how the understanding of pathways regulating liver zonation and niche signals present in the liver microenvironment can be exploited to modulate the hepatocyte expression profile in vitro.

## Modulating hepatocyte function in vitro*:* from the perspective of liver zonation

Hepatocytes within the liver sinusoid are exposed to varying gradients of morphogens, oxygen, nutrients, hormones and metabolites, resulting in zonated gene expression, whereby cells around the portal vein (PV) express different subsets of genes from cells around the CV^112,113^. The WNT/β-catenin pathway is generally considered one of the major determinants of spatial zonation of gene expression and termed the ‘zonation keeper’^114^. Production of WNT9B and RSPO3, as well as WNT2 expressed in sinusoidal endothelial cells extending from the CV, results in a WNT morphogen gradient in the lobule. By contrast, WNT/β-catenin activity is repressed by the negative regulator adenomatous polyposis coli (APC) in the portal region^114^. As Benhamouche et al.^114^ showed, blockade of WNT signaling promotes periportal gene expression, and knocking out *Apc* promotes pericentral gene expression. While WNT ligands are enriched in the pericentral region, RAS signaling has been postulated to be one of the major pathways regulating periportal gene expression (Fig. 2a)^113,115^. In addition to the RAS pathway, glucagon has been shown to regulate liver zonation by opposing the WNT pathway^116^. Likely, many other pathways that may contribute to liver zonation remain to be identified.

In our recent study^25^, we found that the expression levels of zonated genes (e.g., carbamoyl-phosphate synthase 1 [*Cps1*] and *Cyp2f2*) were low in the expansion media but could be modulated in vitro. To induce periportal genes such as *Cps1* and *Cyp2f2*, a medium containing EGF, HGF and DEX (without WNT and TNF-α) was devised. DEX, a corticosteroid that exerts anti-inflammatory effects on hepatocytes, has been previously shown to suppress hepatocyte proliferation and promote differentiation in culture^117^. Another medium containing the WNT agonist CHIR99021 and DEX (without EGF, HGF and TNF-α) was employed to promote pericentral genes such as *Cyp1a2* and glucokinase (*Gck*)^25^. Notably, the expression of these aforementioned genes could be massively induced (up to several thousand-fold) (Fig. 2b). In addition to hepatocytes, nonparenchymal cell types in the liver also display a zonated pattern of expression^118,119^. Halpern et al.^119^ showed, by sequencing closely associated pairs of hepatocytes and endothelial cells, that endothelial cells displayed profound spatial heterogeneity, with one-third of the genes being significantly zonated. For instance, *Wnt9b* and *Rspo3* are enriched in the endothelium of the CV, while *Bmp2* and *Stab1* are repressed. Spatially localized niche signals, either by endothelial cells or other cell types in the liver, likely contribute to the zonated expression pattern of hepatocytes. We anticipate the incorporation of these niche factors in the culture media to improve the expression of zonated genes in hepatocyte organoids.

While it is desirable to recreate liver zonation in vitro to achieve functionally diverse hepatocytes in culture, this has been technically challenging due to the absence of spatially localized niche signals and morphogen gradients typically observed in native tissue. Furthermore, the WNT protein has traditionally been difficult to manipulate due to its insolubility and poor expression yield. Major advances have been made in the field through the engineering of potent soluble WNT agonists^120,121^, which are more amenable to manipulation. WNT agonists are engineered to engage LRP6 and FZD receptors, for instance, by linking a pan-Frizzled antibody to the C-terminal domain of the WNT antagonist DKK1. The use of WNT agonists for culturing various types of epithelial organoids has been established recently^121^. Specifically, we showed that hepatocyte organoids can be robustly expanded using next-generation WNT surrogates, following the protocol described previously^25,121^. Separately, earlier work by Habib et al.^122^ showed that immobilized WNT, i.e., WNT tethered to beads, can act as a stem cell niche to mediate asymmetric division^123,124^. In a recent study, the Lutolf laboratory showed that microstructured biological scaffolds that mimic the crypt-villus structure can be used to maintain the mini-intestine in vitro^125^. Collectively, micropatterned scaffolds, together with coculture with nonparenchymal cell types, tethering of WNT agonists to a ‘signaling center’ mimicking the CV, and modulation of morphogen flow, may enable hepatocyte culture with spatial organization and gradient gene expression that resembles the liver lobule.

## Hepatocyte transplantation to treat liver diseases

Orthotopic liver transplantation is currently the favored treatment option for end-stage chronic liver diseases. Due to severe donor organ shortages, hepatocyte transplantation has been explored as an alternative strategy. Cryopreserved primary human hepatocytes have been used for transplantation in patients with various types of diseases, such as acute liver failure^126^, factor VII deficiency^127^ and Crigler-Najjar syndrome^128–130^ (reviewed by Barahman et al. 2018^131^). However, the lack of good-quality donor cells, limited engraftment efficiency, immune rejection of transplanted cells despite the application of immunosuppression, and the loss of clinical benefit over time have precluded large-scale clinical applications of hepatocyte transplantation. Here, we discuss the types of liver diseases that can potentially be treated by cell transplantation, the potential use of in vitro-expanded hepatocytes for cell transplantation, and strategies to improve their long-term engraftment.

## Types of liver diseases that can potentially be treated by hepatocyte transplantation

**Fig. 3:**
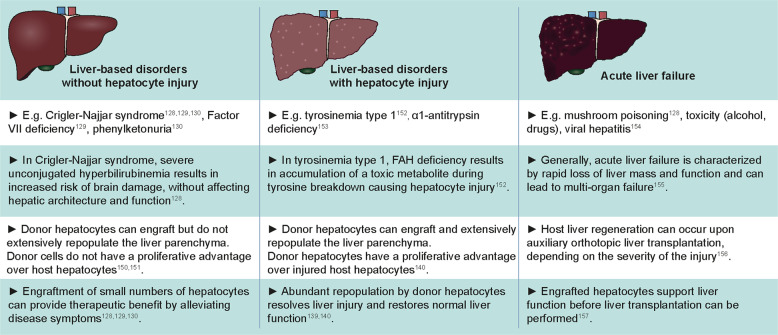
An overview of the types of liver diseases that can potentially be treated with hepatocyte transplantation^128–130,139,140,150–157^. The efficiency of engraftment and repopulation of donor hepatocytes is dictated by the host tissue microenvironment. In addition, the number of donor hepatocytes needed to provide therapeutic benefit is dependent on the liver pathophysiology.

The efficiency of repopulation by hepatocytes in the host liver and the extent of liver repopulation needed for therapeutic benefit vary between different types of liver diseases, depending on the pathophysiology of the diseased host liver (Fig. 3). For instance, many liver-based disorders are caused by a single gene defect, including Crigler-Najjar syndrome^128–130^ (hyperbilirubinemia), factor VII deficiency^127^ (blood clotting disorder) and phenylketonuria^132^ (phenylalanine accumulation in blood). The absence of functional gene products prevents certain essential hepatic functions from being carried out without damaging the liver itself. In this context, small numbers of engrafted hepatocytes can provide therapeutic benefits and alleviate disease symptoms by the production of the necessary functional proteins. The number of engrafted donor cells needed for clinical benefit for these diseases is unknown, but several patients showed reduced disease symptoms after receiving cell numbers corresponding to ~5–10% of the liver mass^131^. For instance, transplantation of ~4% of the total liver mass in a patient with Crigler-Najjar syndrome resulted in greatly decreased serum bilirubin levels, which were maintained for a period of six months, after which disease remission occurred, and the patient eventually received a whole liver transplant^130^. The loss of clinical benefit over time, however, suggests the lack of long-term donor cell engraftment in the host liver.

## Cell transplantation with in vitro-expanded hepatocytes

To improve the clinical outcome of hepatocyte transplantation in humans, major challenges need to be addressed, such as the lack of healthy and good-quality primary cells suitable for transplantation, difficulty in preservation, low engraftment and repopulation and lack of long-lasting effects on the restoration of organ functions^130,131,133^. Cryopreserved primary human hepatocytes have thus far been used for clinical applications of hepatocyte transplantation. While cryopreservation facilitates long-term storage of donor material, cryopreserved hepatocytes showed reduced cell viability, cell attachment efficiency and albumin production compared to freshly isolated primary hepatocytes, leading to compromised quality for cell transplantation^133^. Cultured hepatocytes tolerate cryopreservation better than freshly isolated primary hepatocytes. Rodent hepatocytes that were cultured for 24 h in a monolayer prior to cryopreservation displayed higher viability and plating efficiencies upon thawing than primary hepatocytes that were cryopreserved directly after isolation^134^. Based on our own experiences, hepatocyte organoids can be freeze-thawed repeatedly for in vitro expansion. Cultured hepatocytes^25^ could be shipped overnight on ice in expansion medium supplemented with 2% fetal bovine serum and retained high viability (>90% viability, our unpublished data) for transplantation in mice.

## Engraftment of in vitro-expanded primary hepatocytes in liver injury mouse models

### Cholangiocyte-derived organoids

Huch et al.^20^ showed that biliary organoids derived from intrahepatic cholangiocytes could be induced to express hepatocyte markers (e.g., HNF-4α, ALB and multidrug resistance-associated protein 4 (MRP4)) and display some hepatic functions (e.g., LDL uptake and glycogen storage) in vitro. However, these ‘transdifferentiated’ biliary organoids showed limited repopulation in fumarylacetoacetate hydrolase-deficient (*Fah*^−/−^) mice (< 1% of the liver parenchyma)^20^. Similarly, human biliary organoids only engrafted as singlets and doublets into the mouse liver and showed no evidence of repopulation in BALB/c nude mice^21^. Such low repopulation of the liver by biliary organoids is most likely a result of poor transdifferentiation of biliary cells to hepatocytes in vitro prior to transplantation and/or in vivo following transplantation. In line with this, cholangiocyte to hepatocyte transdifferentiation is rarely observed during liver regeneration in vivo^135,136^ but only under extreme circumstances where hepatocyte proliferation is severely impaired in genetically modified mouse models^137^. The lack of engraftment and repopulation using cholangiocyte-derived organoids demonstrated the need for using primary hepatocyte organoids as the cell type for transplantation.

### Primary hepatocyte organoids

The in vivo regenerative capacity of in vitro-expanded primary hepatocytes was demonstrated by transplantation of murine hepatocyte organoids into *Fah*^−/−^ mice^25^. Engrafted hepatocytes massively repopulated the injured host liver at approximately 100 days posttransplantation, resulting in occupation of up to 80% of the liver parenchyma by FAH^+^ clones of various sizes. Donor cells expressed the hepatocyte marker HNF-4α but not the biliary markers SOX9, KRT7 or KRT19. Of note, hepatocyte organoids were cultured in media promoting expansion prior to transplantation. Intriguingly, donor cells underwent maturation in vivo and reestablished expression of zonation markers. For instance, cells adjacent to the CV expressed markers such as glutamine synthetase (GS), glutamate transporter 1 (GLT1) and CYP2E1, while cells outside the CV areas expressed CPS1. Notably, the expression of these markers was not detected in the host tissue^25^. Overall, these observations suggest that the expression profile of donor hepatocytes upon transplantation is dictated by the host liver microenvironment.

### Hepatocyte organoids derived from human fetal liver

Hu et al.^26^ were able to successfully engraft human fetal liver-derived hepatocyte organoids in *Fah*^−/−^*/Rag2*^−/−^*/Il-2rγ*^−/−^ (FRG)N mice (i.e., FRG-deficient mice in a nonobese diabetic (NOD) strain), albeit to a limited extent. Nonetheless, engrafted cells expressed functional markers, such as ALB, CYP2E1 and MRP2, but not the fetal marker AFP, indicative of in vivo maturation. The reason for the limited extent of engraftment is unclear and could be attributed to the lack of maturation in vivo. To test this hypothesis, the authors compared the engraftment of fetal-derived organoids with primary hepatocytes and organoids obtained from one pediatric source and noted that primary hepatocytes and organoids significantly outperformed fetal-derived organoids in terms of engraftment and proliferation^26^. This result suggests that mature hepatocytes are better suited for cell transplantation.

### 2D culture of primary murine/human hepatocytes

In addition to in vitro-expanded primary hepatocyte organoids, an earlier study by Katsuda et al.^75,100^ showed that chemically induced liver progenitors (CLiPs) generated from rat hepatocytes retained high in vivo repopulation capacity. Using cDNA-uPA/severe combined immunodeficient (SCID) mice, which express uPA specifically in hepatocytes under the albumin promotor to induce severe liver injury, CLiPs repopulated up to 90% of the host livers and expressed HNF-4α, multidrug resistance-associated protein 2 (MRP2) and various CYP enzymes, indicative of substantial in vivo differentiation^75,100^. Building upon their previous work^75^, Katsuda et al.^100^ showed that human infant hepatocytes reprogrammed in culture to human CLiPs displayed highly variable repopulation efficiency in TK-NOG and cDNA-uPA/SCID mice. In some mice, repopulation of more than ~90% of the liver parenchyma was achieved, and cells expressed the functional markers MDR1, TTR, GS, CYP1A2, and CYP3A4, indicating maturation in vivo. In another study, Zhang et al.^96^ showed that cryopreserved human hepatocytes that were expanded in vitro could substantially repopulate the livers of FRG mice. Donor cells expressed the hepatocyte markers HNF-4α, ALB, CYP3A4, and GS^96^. Notably, the latter two studies showed that the repopulation capacity of expanded human hepatocytes decreased upon longer in vitro culture, as cells at later passages showed reduced repopulation after transplantation when compared to earlier passages^96,100^. These studies suggest that suboptimal culture conditions may have somewhat compromised the engraftment potential of the cells.

## Regenerative host liver microenvironment facilitates donor cell repopulation

**Fig. 4:**
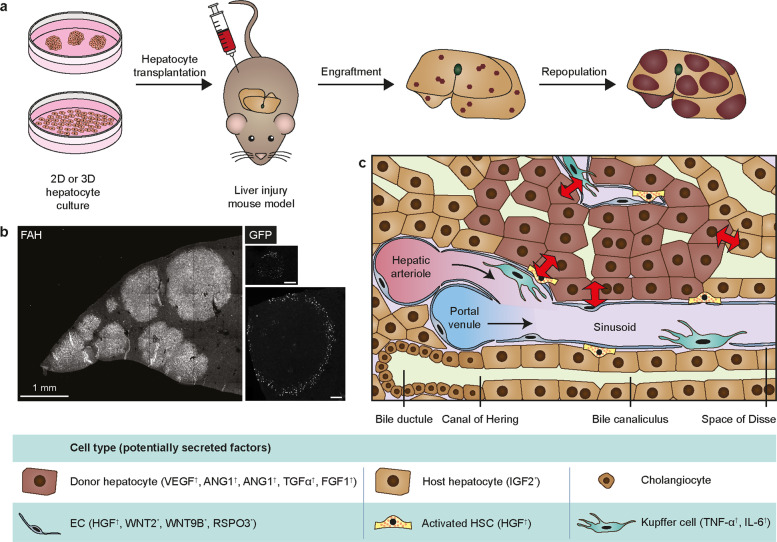
An overview of hepatocyte transplantation in mice and cellular crosstalk between donor hepatocytes and the host liver microenvironment. **a** Preclinical hepatocyte transplantation studies are typically performed in mice with inducible hepatocyte-specific liver injury to evaluate the in vivo regenerative capacity of in vitro-expanded hepatocytes. In this setting, the regeneration of host hepatocytes is impaired, giving donor cells a proliferative advantage. **b** Transplantation of cultured murine hepatocytes in *Fah*^−/−^ mice results in repopulation of the host liver parenchyma by donor cells. Left panel: Transplanted murine hepatocyte organoids gave rise to grafts of various sizes, as shown by immunostaining of FAH. Right panels: upon transplantation of hepatocytes isolated from doxycycline-treated *Axin2-rtTA;TetO-H2B:GFP* reporter mice, GFP^+^ cells could be observed in donor cell grafts by immunofluorescent imaging. Upper panel: smaller grafts showed scattered GFP^+^ donor cells. Bottom panel: Larger grafts showed GFP^+^ cells at the graft-host tissue boundary only. Scale bars: 100 µm. Images adapted from Peng et al. 2018^25^. **c** Crosstalk between donor hepatocytes, host hepatocytes, and other cell types in the injured host liver microenvironment facilitates donor cell repopulation. Potential factors that contribute to donor cell repopulation in the host liver, based on hepatocyte transplantation studies^25,144–147^ (indicated by ^*^) and liver regeneration studies^53,60,63,142^ (^†^), are listed in the lower panel. ANG: angiopoietin; HSC: hepatic stellate cell; EC: endothelial cell.

How hepatocytes repopulate the host liver remains an intriguing question. Previous studies by the Grompe laboratory and others demonstrated that transplanted primary hepatocytes could replace up to 90% of the mouse host liver of *Fah*-deficient^138^ or FRG-deficient^139^ mice, which are widely used to study liver repopulation (Fig. 4a). Given that ~1 million cells (or < 2% of total liver mass) are typically transplanted into mice and only ~10% of the injected cells are estimated to engraft in the liver^139^, significant expansion in vivo needs to occur to achieve the levels of repopulation observed. In these studies, *Fah*^−/−^ mice were continuously administered the drug 2-(2-nitro-4-trifluoro-methylbenzyol)-1,3-cyclohexanedione (NTBC) in drinking water since birth to prevent the production of toxic metabolites and the onset of liver injury. Upon transplantation, NTBC is withdrawn to initiate liver injury^140^. In *Fah*^−/−^ mice, the regeneration of host hepatocytes, but not of donor cells, is impaired, which provides donor cells with a proliferative advantage^140^. Presumably, the regenerative signal-rich liver microenvironment facilitates the proliferation of donor cells.

In *Fah*^−/−^ mice transplanted with hepatocyte organoids, FAH^+^ grafts of various sizes were observed, which most likely arose from clonal repopulation of engrafted cells (Fig. 4b)^25^. To track WNT activity in donor cells, hepatocytes derived from mice carrying the *Axin2-rtTA;TetO-H2B:GFP* reporter gene were transplanted into *Fah*^−/−^ mice. Two days prior to analysis, transplanted mice were administered doxycycline to activate *Axin2* reporter expression in donor hepatocytes. GFP expression could be detected in the FAH^+^ clones but not host cells. In smaller FAH^+^ clones, many GFP^+^ cells were observed scattered throughout the clones, indicating a response to WNT signaling (Fig. 4b). In contrast, in larger grafts, GFP^+^ cells were restricted to donor cells at the graft-host tissue boundary and were absent from the center of the grafts (Fig. 4b). Consistent with this observation, the expression of *Wnt2*, *Wnt9b*, and *Rspo3* was largely upregulated in host tissue^25^. Expression of RSPO3, which is normally restricted to endothelial cells around CVs in uninjured livers^33,34^, was upregulated in endothelial cells in the host tissue around the clonal boundary^25^. Likely, the WNT-rich environment in the host liver facilitates donor cell proliferation, consistent with the role of WNT signaling in other regeneration models^141^.

Previous studies have shown that angiogenesis is a critical aspect of liver regeneration and function^109,110,142^. VEGF secreted by regenerating hepatocytes promotes LSEC proliferation. Neutralization of VEGF not only impaired the proliferation of endothelial cells but also indirectly impaired hepatocytes^142^. Further, it was demonstrated that LSECs establish an inductive vascular niche during liver regeneration after partial hepatectomy through activation of VEGF receptor 2 (VEGFR2) and upregulation of transcription factor inhibitor of DNA binding 1 (ID1), which results in the production of WNT2 and HGF to stimulate hepatocyte proliferation^143^. These observations are consistent with the crosstalk between hepatocytes and LSECs, which serve as a major source of WNT-RSPO signals. In addition to endothelial cell-hepatocyte crosstalk, another example of paracrine signaling between host tissue and donor hepatocytes has been described. Upon transplantation of donor hepatocytes, IGF2 was upregulated by host hepatocytes to support liver repopulation. While blocking IGF2 in vivo impaired liver regeneration, its activation resulted in increased hepatocyte engraftment and numbers of MKI67-positive donor cells^144,145^. It is likely that a network of crosstalk between donor hepatocytes, host hepatocytes, and the host microenvironment dictates repopulation by donor hepatocytes (Fig. 4c).

The contribution of HSCs to hepatocyte engraftment has been studied in a preclinical transplantation setting using animal models that do not allow for selective repopulation of donor cells. Upon hepatocyte transplantation in DPPIV-deficient rats, increased numbers of host HSCs were observed posttransplantation, primarily in close proximity to engrafted DPPIV^+^ donor cells, suggesting close interaction and potential crosstalk between HSCs and donor hepatocytes during donor cell engraftment^146^. In a later study, primary human hepatocytes were cotransplanted with isolated human HSCs or an HSC cell line (LX-2) into SCID mice^147^. Transplantation of human hepatocytes alone resulted in engraftment of scattered human ALB^+^ cells throughout the parenchyma, whereas cotransplantation resulted in increased engraftment, which was more evident for LX-2 cells and culture-activated HSCs than for quiescent HSCs^147^. Together, these studies suggest that a close interaction between hepatocytes and HSCs is involved in the regulation of hepatocyte engraftment upon transplantation, although the exact mechanism and signaling pathways involved and the question of whether HSCs also contribute to supporting liver repopulation by donor cells remain to be elucidated. Although the role of HSCs during liver regeneration is not well understood, it is known that HSCs are activated in response to paracrine signals produced by regenerating hepatocytes and other cell types in the microenvironment (Kupffer cells and LSECs). In addition, HSCs are a main source of HGF, a driving mitogenic factor of hepatocyte proliferation for liver regeneration upon partial hepatectomy^63^. Possibly, similar crosstalk between HSCs and donor hepatocytes also contributes to donor cell engraftment and repopulation.

The role of Kupffer cells and other immune cells in hepatocyte transplantation is complicated, as they are largely involved in the clearance of transplanted cells and prevention of engraftment, which is beyond the scope of this review and has been discussed elsewhere^148^. On the other hand, Kupffer cells are known to be a prominent source of TNF-α and IL-6, two major inflammatory cytokines that are involved in liver regeneration^63,78–80^ and support the proliferation of hepatocytes in vitro^25,81,86^. Therefore, a supporting role for Kupffer cells during liver repopulation by transplanted hepatocytes in vivo could also be expected.

## Conclusions

**Fig. 5:**
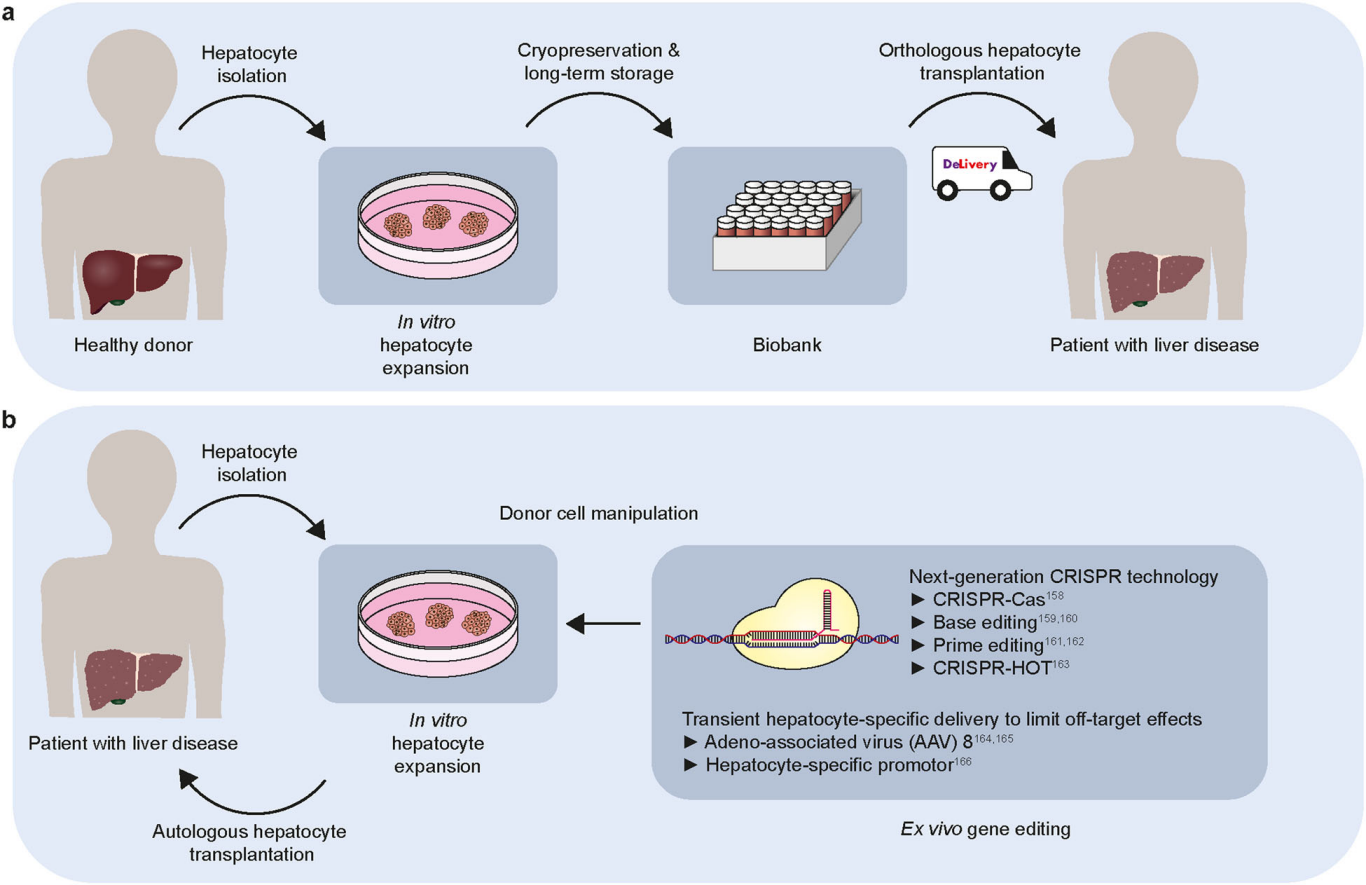
Cell replacement therapy using in vitro-expanded hepatocytes to treat liver diseases. **a** Isolated primary hepatocytes from healthy donors can be expanded in vitro and cryopreserved in a biobank to provide a large supply of high-quality donor material. Biobank material can be employed for orthologous hepatocyte transplantation in patients with liver diseases. **b** Novel gene editing techniques^158–163^ and hepatocyte-specific delivery methods^164–166^ can be applied to patient-derived hepatocytes for ex vivo gene editing to enable autologous hepatocyte transplantation.

The ability to generate or expand the appropriate cell type in vitro, thus providing an abundant supply of healthy donor cells, is the first essential step in making cell replacement therapy feasible. Recent breakthroughs in expanding primary hepatocytes in vitro while retaining their in vivo regenerative capacity represent a major milestone towards enabling hepatocyte transplantation. Furthermore, the ability to expand, cryopreserve and transport hepatocytes without compromising their quality would enable the creation of a biobank that would ensure the availability of (in vitro-expanded) donor materials for hepatocyte transplantation, thus resolving the issue of donor shortage (Fig. 5a). More importantly, autologous cell transplantation will become feasible, as novel gene editing techniques and hepatocyte-specific delivery methods can be applied to patient-derived hepatocytes for ex vivo gene editing to restore disease-specific mutations (Fig. 5b). Although currently, the lack of long-term efficacy due to either insufficient engraftment or lack of long-term maintenance of donor cells in host livers may pose a major challenge in cell transplantation, we expect the understanding of the mechanisms through which donor hepatocytes repopulate the diseased host liver to provide insights into strategies for overcoming these limitations (Fig. 5b). We are optimistic that in vitro-expanded primary hepatocytes will be widely employed for cell replacement therapy to treat liver diseases in the foreseeable future.
